# Author Correction: Structure of a nucleosome-bound MuvB transcription factor complex reveals DNA remodelling

**DOI:** 10.1038/s41467-023-36061-7

**Published:** 2023-01-19

**Authors:** Marios G. Koliopoulos, Reyhan Muhammad, Theodoros I. Roumeliotis, Fabienne Beuron, Jyoti S. Choudhary, Claudio Alfieri

**Affiliations:** 1 Division of Structural Biology, Chester Beatty Laboratories, The Institute of Cancer Research, London, UK; 2 Functional Proteomics, Chester Beatty Laboratories, Cancer Biology Division, The Institute of Cancer Research, London, UK

**Keywords:** Cryoelectron microscopy, DNA

Correction to: *Nature Communications* 10.1038/s41467-022-32798-9, published online 29 August 2022

The Supplementary Information file in the original version of this Article was missing Supplementary Table 1. This has now been corrected in both the PDF and HTML versions of the Article. In addition, the Data Availability Statement in the original version of this Article has been edited to clarify which complex structure maps have been deposited to the PDB and the EMDB.

## Supplementary information

Updated Supplementary Information

