# Supplementary material for: Structure of a nucleosome-bound MuvB transcription factor complex reveals DNA remodelling
Source: Nat Commun. 2022 Aug 29;13:5075. doi: 10.1038/s41467-022-32798-9 (PMC9424243; doi:10.1038/s41467-022-32798-9)
Supplement: Supplementary file 1 — Updated Supplementary Information [file 41467_2022_32798_MOESM1_ESM.pdf]

Supplementary Information

Structure of a nucleosome-bound MuvB transcription factor complex reveals DNA remodelling

Marios G. Koliopoulos[#1], Reyhan Muhammad[#1], Theodoros I. Roumeliotis[2], Fabienne Beuron[1], Jyoti S. Choudhary[2], and Claudio Alfieri[1*]

[#]Contributed equally

[1]Division of Structural Biology, Chester Beatty Laboratories, The Institute of Cancer Research, London, UK.

[2]Functional Proteomics, Chester Beatty Laboratories, Cancer Biology Division, The Institute of Cancer Research, London, UK.

[*]Corresponding author. Tel: +44 20715 35087; E-mail: claudio.alfieri@icr.ac.uk

Supplementary Figure 1

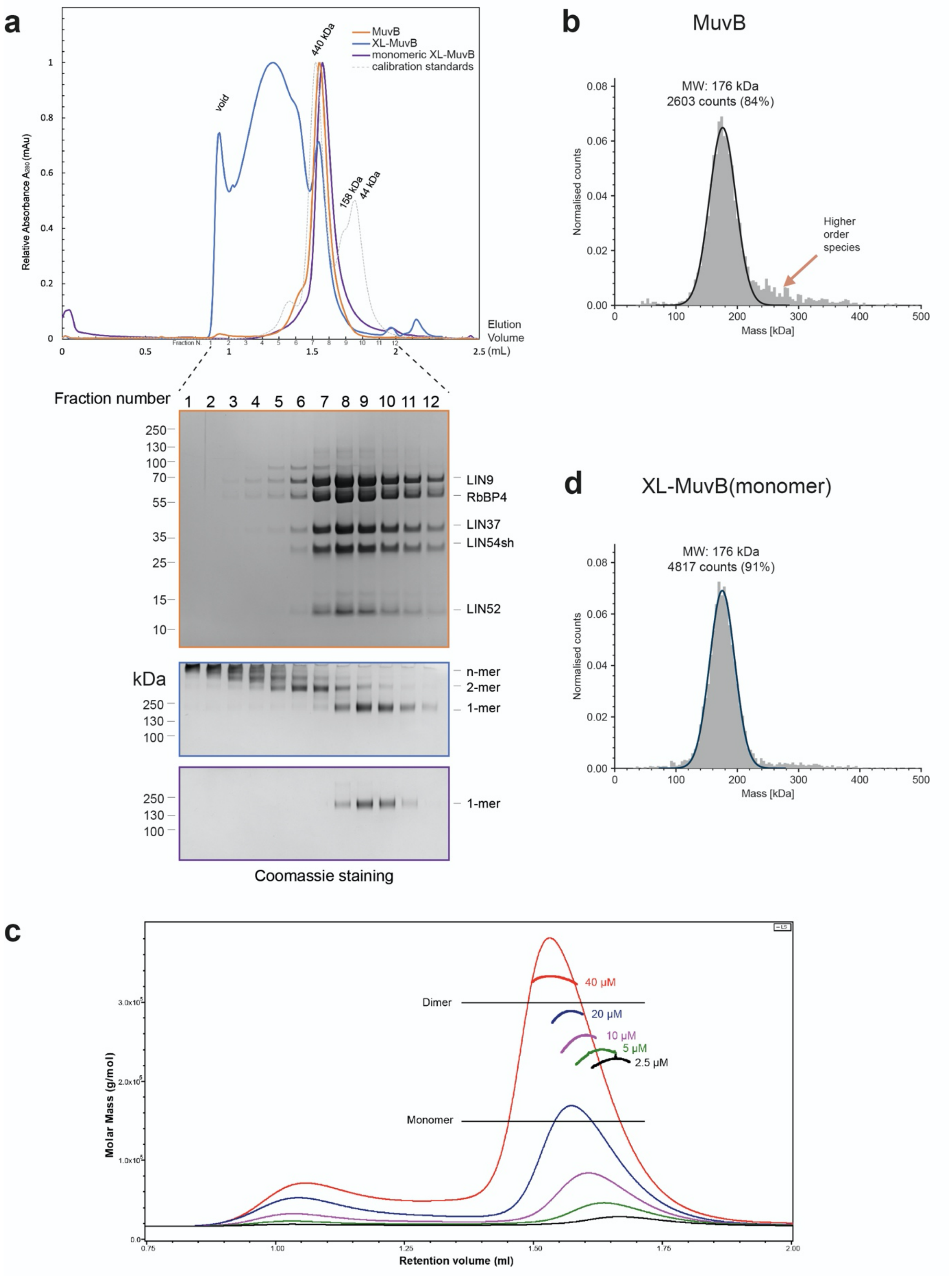

Supplementary Fig. 1. Biochemical and biophysical characterisation of the MuvB complex. a Size exclusion chromatography (SEC) chromatogram (Top) of the MuvB complex without or with crosslinking treatment (XL-MuvB) prior to the run. Calibration standards are

illustrated for references of molecular weight. Coomassie stained gel of the eluted fractions (Bottom). Colour code indicates the different sample in the gel. These experiments were repeated independently three times with similar results. **b** Mass photometry analysis of MuvB. Theoretical mass for a 1:1:1:1:1 complex (LIN9, RbBP4, LIN37, LIN52, LIN54sh(a.a. 515-749)) is 176.7 kDa. **c** SEC MALS analysis of MuvB^{core} at indicated concentrations. Theoretical mass for a 1:1:1:1 complex (LIN9, RbBP4, LIN37, LIN52) is 150.9 kDa. **d** Mass photometry of XL-MuvB after removal of oligomers (XL-MuvB monomer) shows a higher level of homogeneity that was necessary for further cryo-EM analysis.

Supplementary Figure 2

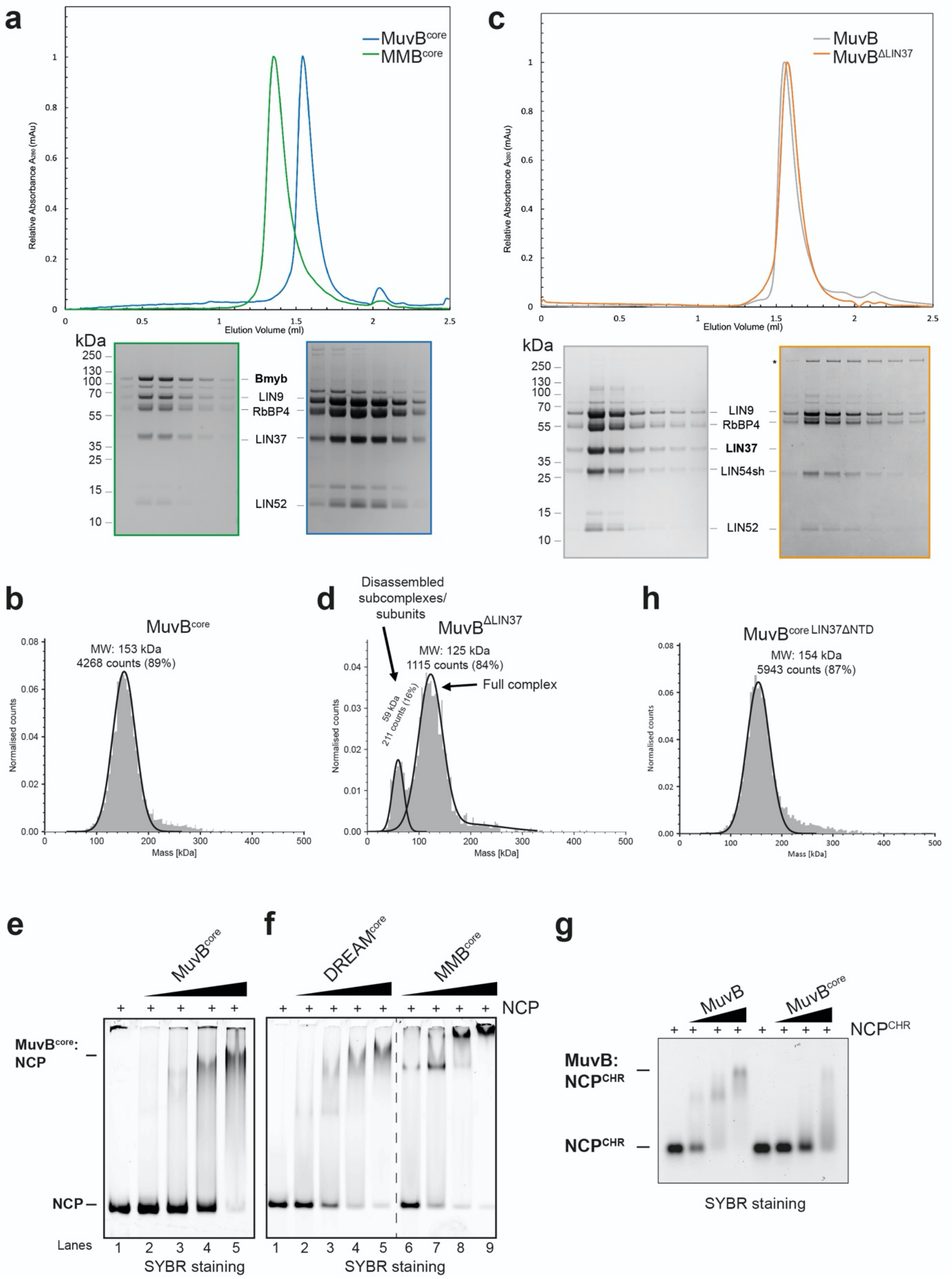

Supplementary Fig. 2. Biochemical and biophysical characterisation of MuvB subcomplexes. a-d SEC (**a**) and mass photometry data (**b**) for MuvB^{core} and MuvB ΔLIN37 (**c-d**) subcomplexes are shown as in Supplementary Fig. 1. Theoretical mass for a MuvB^{core}

1:1:1:1 complex (LIN9, RbBP4, LIN37, LIN52) is 150.9 kDa. Theoretical mass for a 1:1:1:1 MuvB^{core} ΔLIN37 complex composed by LIN9, RbBP4, LIN52 and LIN54sh (a.a. 515-749) is 148.4 kDa. These experiments were repeated independently three times with similar results. **e** Electrophoretic mobility shift assay (EMSA) of 167 nucleosome (NCP) with distinct MuvB complexes and subcomplexes used in this study. The MMB^{core} complex shows a sharper shifted band as a sign of better homogeneity of the complex formed. For MuvB^{core} EMSA (**e**), NCP:MuvB^{core} molar ratios are 1:1, 1:2, 1:4, 1:6. For **f** DREAM^{core} (containing MuvB^{core}:retinoblastoma-like 2 protein complex) and MMB^{core} EMSA, NCP:protein molar ratios are 1:1, 1:2, 1:3, 1:4. **g** EMSA of CHR- containing NCP with either MuvB containing LIN54sh or MuvB^{core}. NCP^{CHR}:MuvB^{core} molar ratios are 1:1, 1:2, 1:4. **e-f** are PAGE gels **g** is an agarose gel. **h** Mass photometry data for the MuvB^{core} LIN37ΔNTD mutant. Each of the experiments in **e**, **f** and **g** were repeated independently three times with similar results.

Supplementary Figure 3

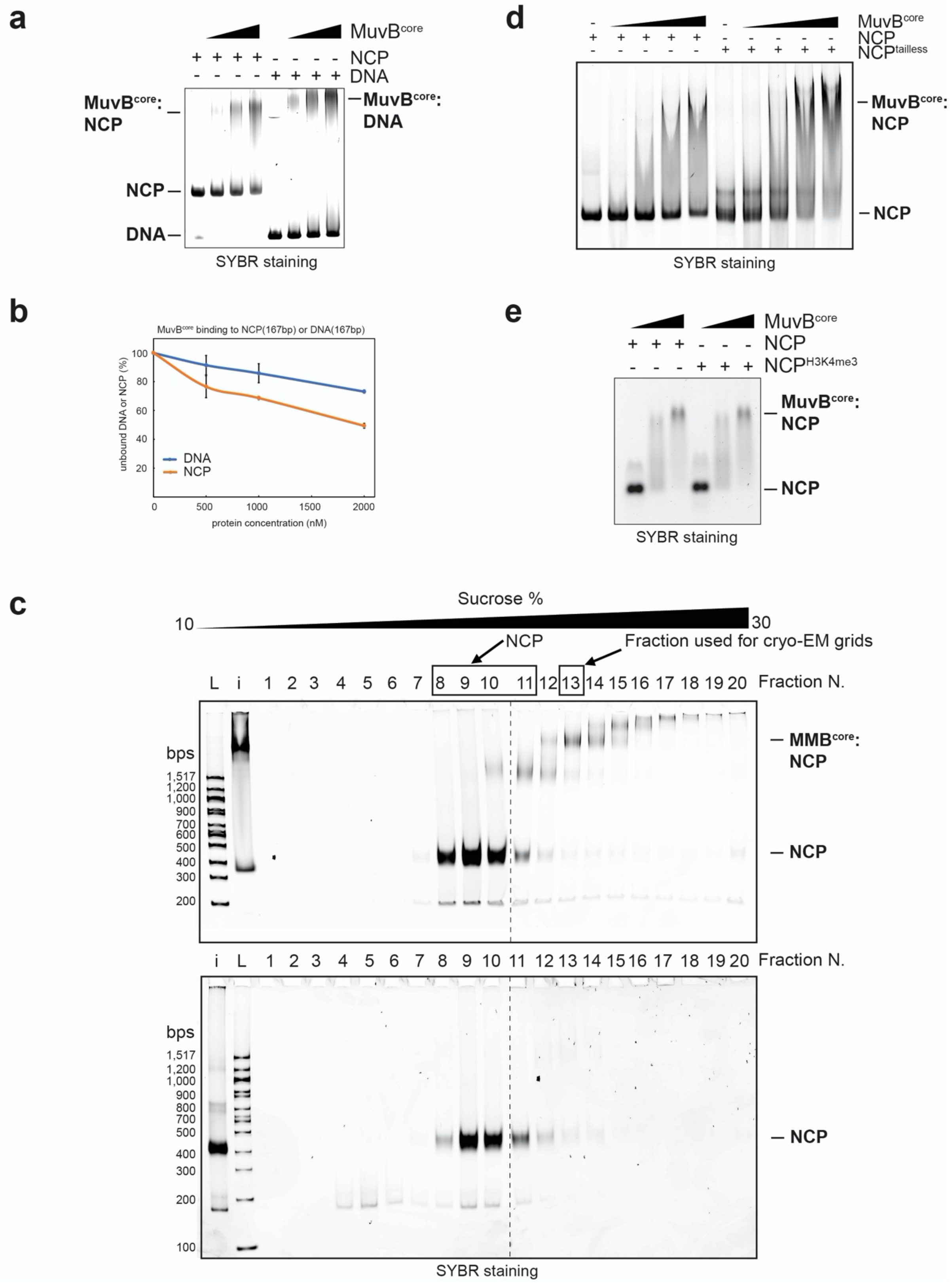

Supplementary Fig. 3. Formation of MuvB complexes with the nucleosome (NCP). a EMSA of either 167 nucleosome (NCP) or free 167 DNA performed with MuvB^{core}. Molar ratios are 1:1, 1:2, 1:4. Quantification data of the bands in this gel are plotted in **b**. Data are

presented as mean values ± standard error of the mean of three independent experiments (n= 3). From this experiment, the affinity is in the low μM range with the nucleosome complex binding ~1.8 folds better. **c** Native gel run with GraFIX elutions from the MuvB^{core}:NCP complex. Sucrose concentrations defining the sucrose gradient are indicated at the top. Fraction N. 13 presents only one species, as a sign of homogeneity, and it is well separated from the free NCP (fractions N. 8-11). Fraction 13 was then used for cryo-EM grid analysis. **d** EMSA of either 167 nucleosome (NCP) or tailless (NCP^{tailless}) 167 nucleosome performed with MuvB^{core}. Molar ratios are 1:1, 1:2, 1:3 and 1:4. **e** EMSA of either 167 nucleosome (NCP) or H3K4me3 nucleosome (NCP^{H3K4me3}) performed with MuvB^{core}. Molar ratios are 1:1, 1:2, 1:4. **a,c-d** are PAGE gels **e** is an agarose gel. The experiments in **c**, **d** and **e** were repeated independently three times with similar results.

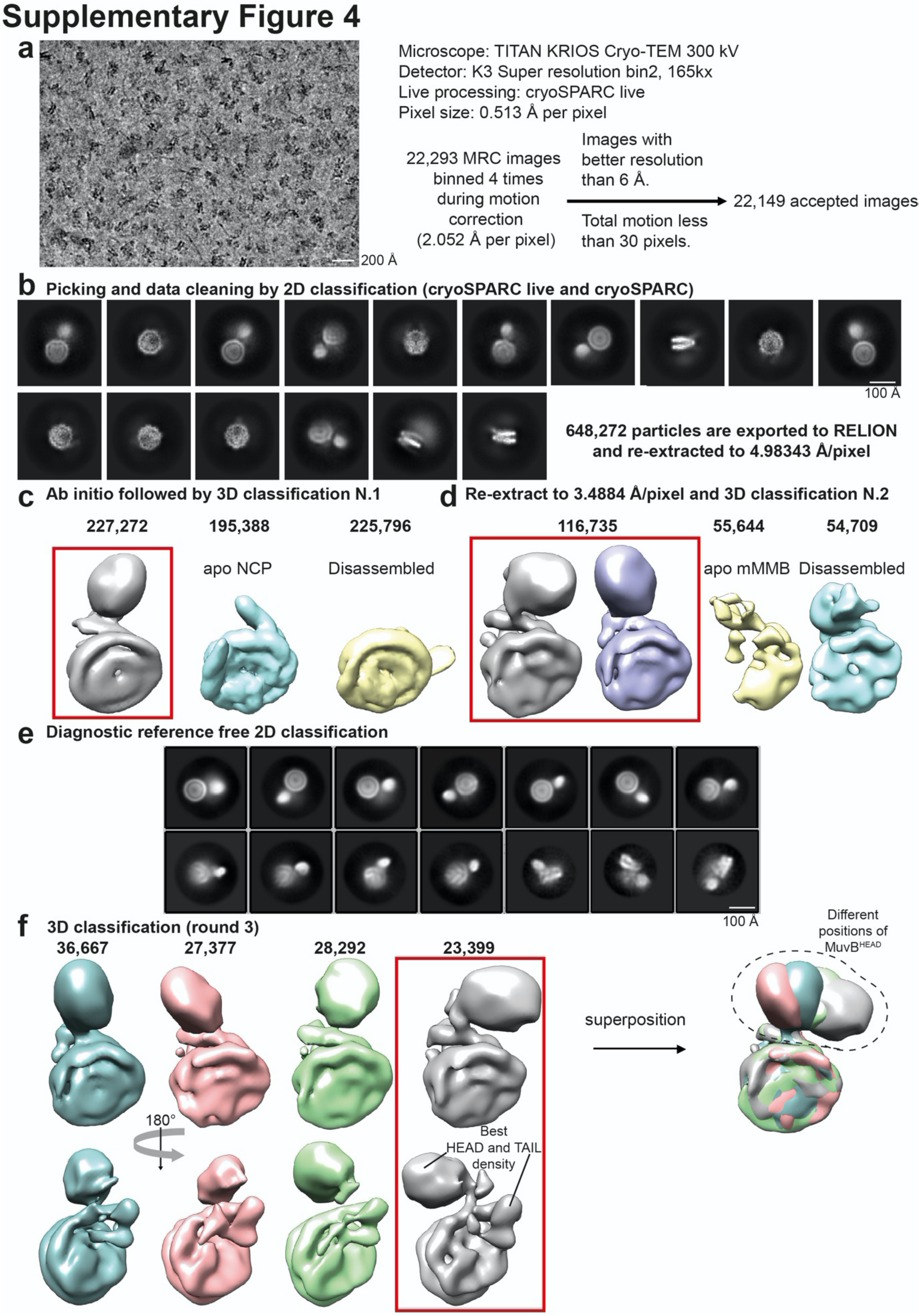

Supplementary Fig. 4. Cryo-EM analysis of MMB^{core}:NCP complex (part 1). a-f Workflow showing a representative micrograph (low-pass filtered to 15 Å), the cryo-EM data collection

parameters (**a**) and the single-particle analysis pipeline (**b-f**). N. of particles at each classification step is indicated. More details are described in the main text and in the Methods section.

Supplementary Figure 5

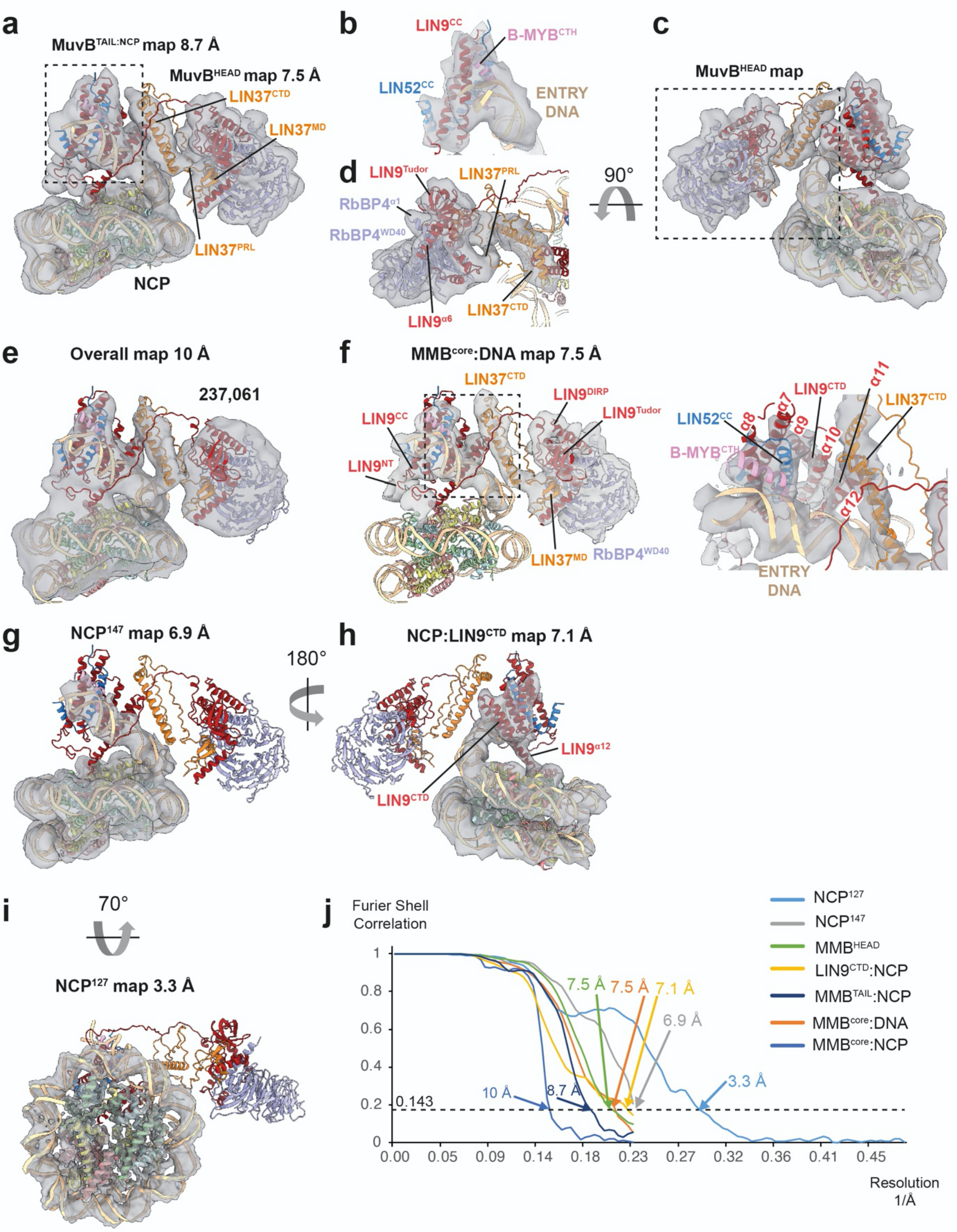

Supplementary Fig. 5. Cryo-EM analysis of MMB^{core}:NCP complex (part 2). a-d Maps obtained by focused 3D refinements on the selected class from Supplementary Fig. 4f. Two views related by a 180 degrees rotation on the y-axis of the MuvB^{TAIL}:NCP and MuvB^{HEAD} maps with the respective fitted coordinates are shown in **a** and **c**. **b** Zoomed view on the LIN9:52CC:B-MYB^{CTH}:DNA quaternary assembly from **a**. **d** Zoomed view on the MuvB^{HEAD} assembly from **c**. **e** Overall map of the MMB^{core}:NCP structure obtained from merging particles of 2 datasets collected (see Methods). The number of particles used is indicated. **f-i** Maps obtained by focused 3D refinements performed on **e**. **j** Fourier Shell Correlation (FSC) curves for all the reconstructions are shown.

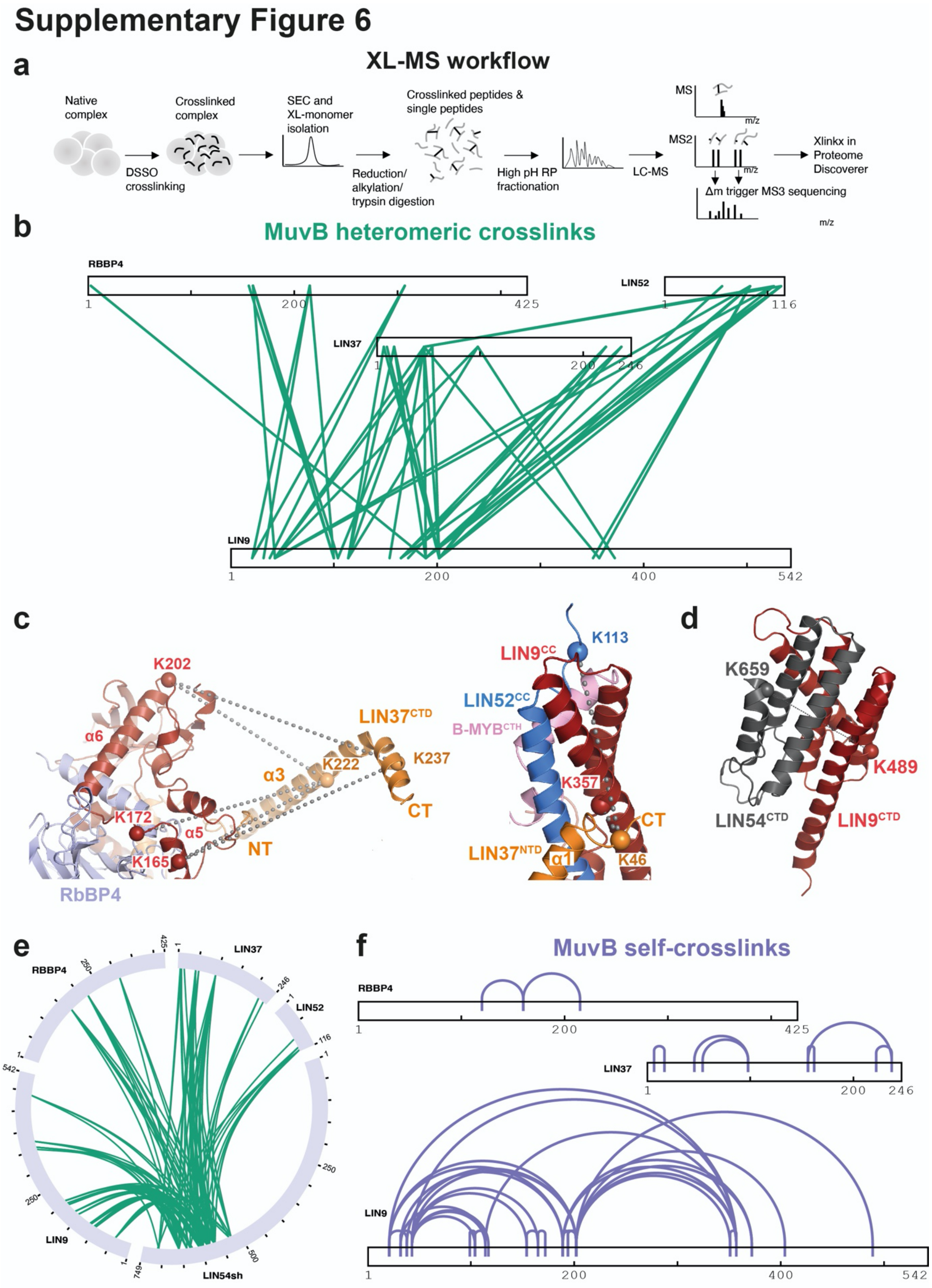

Supplementary Fig. 6. XL-MS workflow applied to the MuvB apo complex. a Workflow performed for the XL-MS analysis of the apo MuvB complex, composed of LIN9, RbBP4, LIN37, LIN52, and LIN54sh (a.a. 515-749). An MS-cleavable crosslinker was used (DSSO)

followed by proteolysis, peptide fractionation and an MS2-MS3 acquisition strategy. Detected XLinked peptides are also shown on Supplementary Data 1. To obtain high confidence data for model interpretation and visualization, only the Xlinks to a value of >100 were used. **b** Heteromeric crosslinks were visualised with xiVIEW (https://xiview.org/xiNET_website). **c** MuvB heteromeric crosslinks from the XL-MS data on MuvB are displayed on our 3D model. These crosslinks support proximity of LIN37^{CTD} and LIN37^{NTD} in respect to the LIN9^{DIRP} and the LIN9:LIN52CC respectively. **d** AlphaFold model of the LIN9^{CTD}:LIN54^{CTD} subcomplex, this assembly is supported by our crosslinking data. The AlphaFold prediction overall has high score. **e** Heteromeric crosslinks of LIN54 with the other MuvB subunits. **f** Self-crosslinks shown in the MuvB subunits LIN9, RbBP4, and LIN37.

Supplementary Figure 7

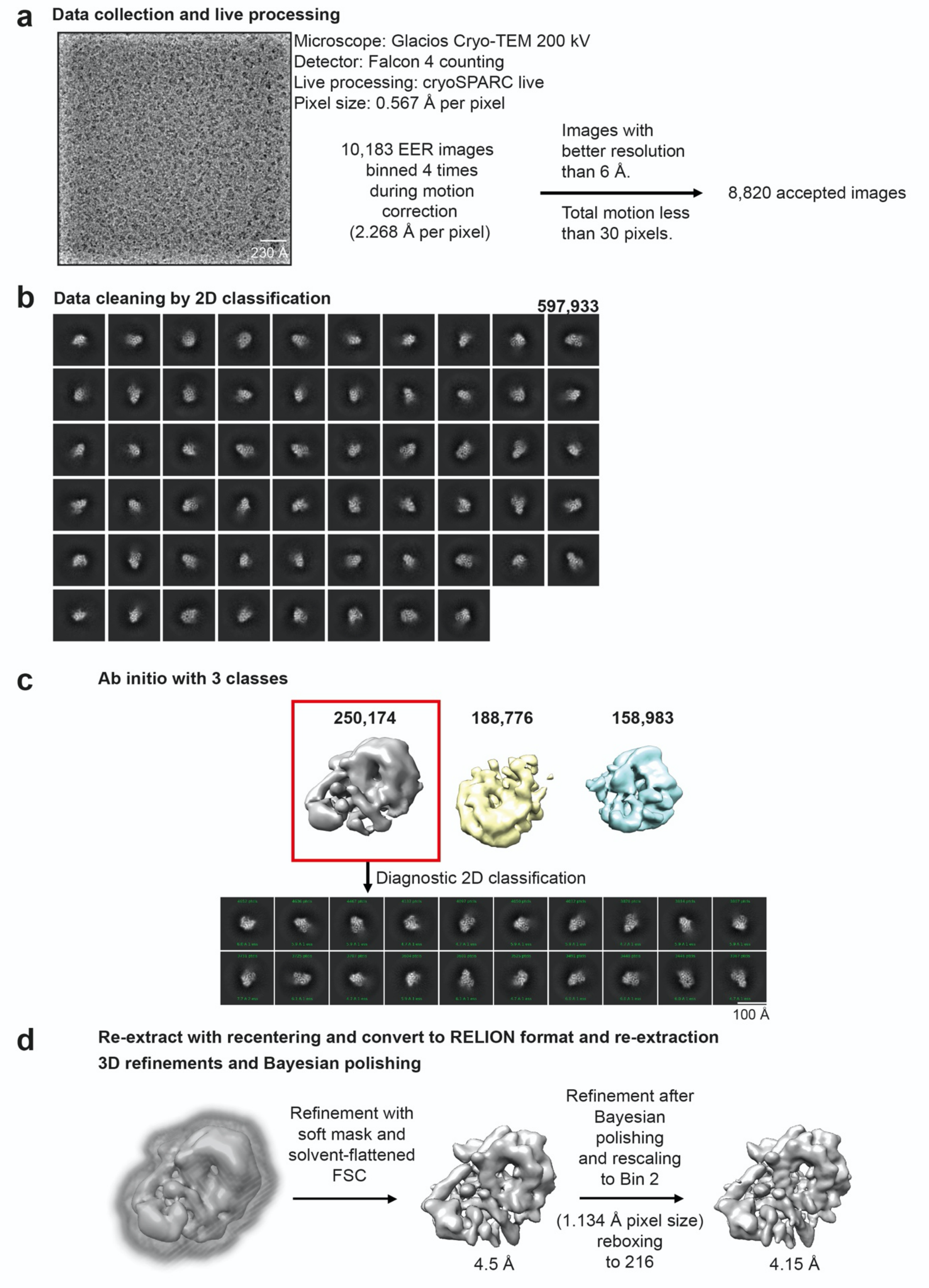

Supplementary Fig. 7. Cryo-EM analysis of the MuvB apo complex (part 1). a-d Workflow showing a representative cryo-EM micrograph (low-pass filtered to 15 Å), cryo-EM data collection parameters (**a**) and the single-particle analysis pipeline (**b-d**). Number of particles at each classification step is indicated. More details are described in the Methods section.

Supplementary Figure 8

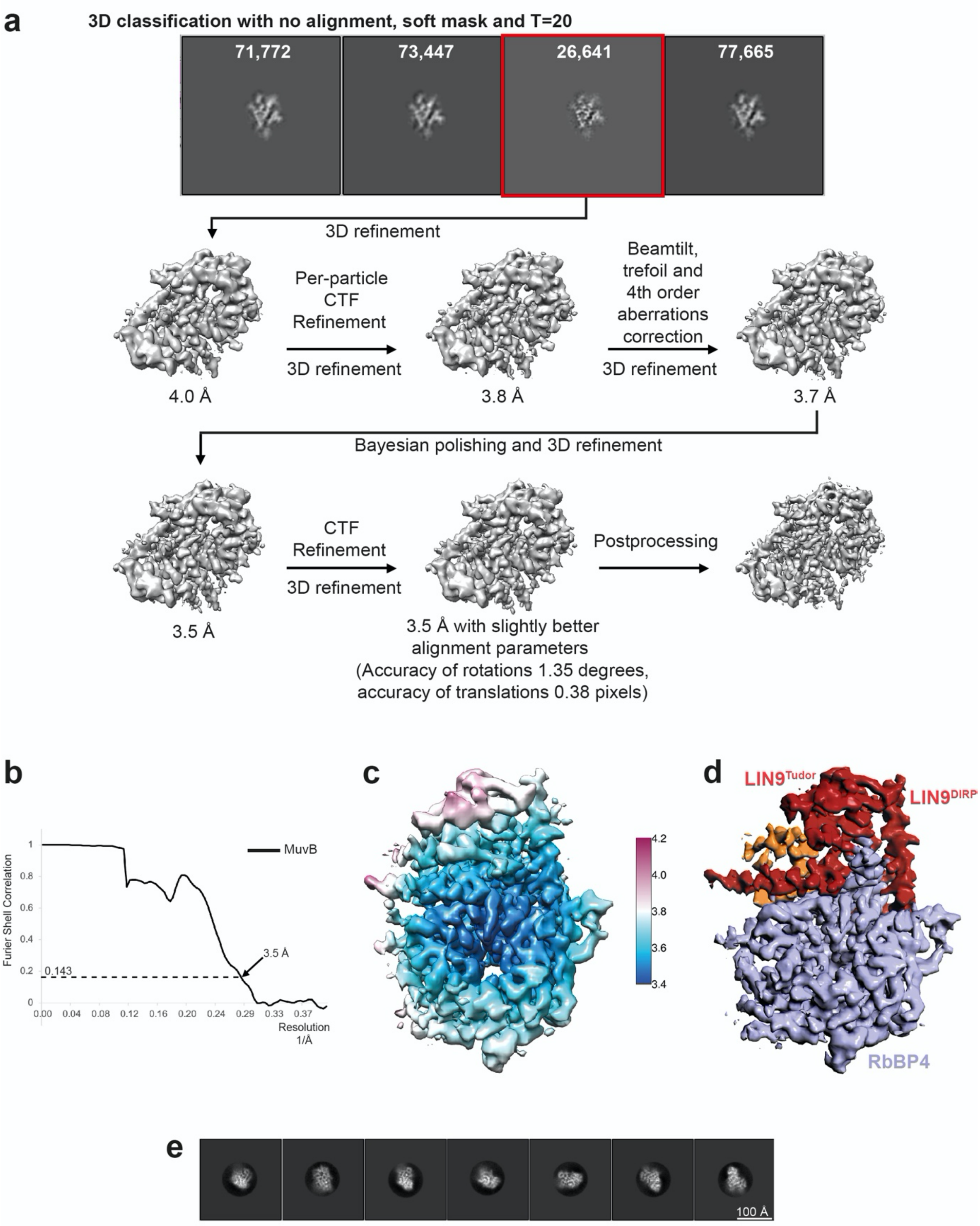

Supplementary Fig. 8. Cryo-EM analysis of the MuvB apo complex (part 2). a the single-particle analysis pipeline (continuation from Supplementary Fig. 7) is indicated (more details are explained in the Methods section). Fourier Shell Correlation (FSC) curve (**b**), local resolution map calculated with RELION (**c**), map coloured by subunit (**d**), final 2D class averages (**e**) are shown for the MuvB complex reconstruction.

Supplementary Figure 9

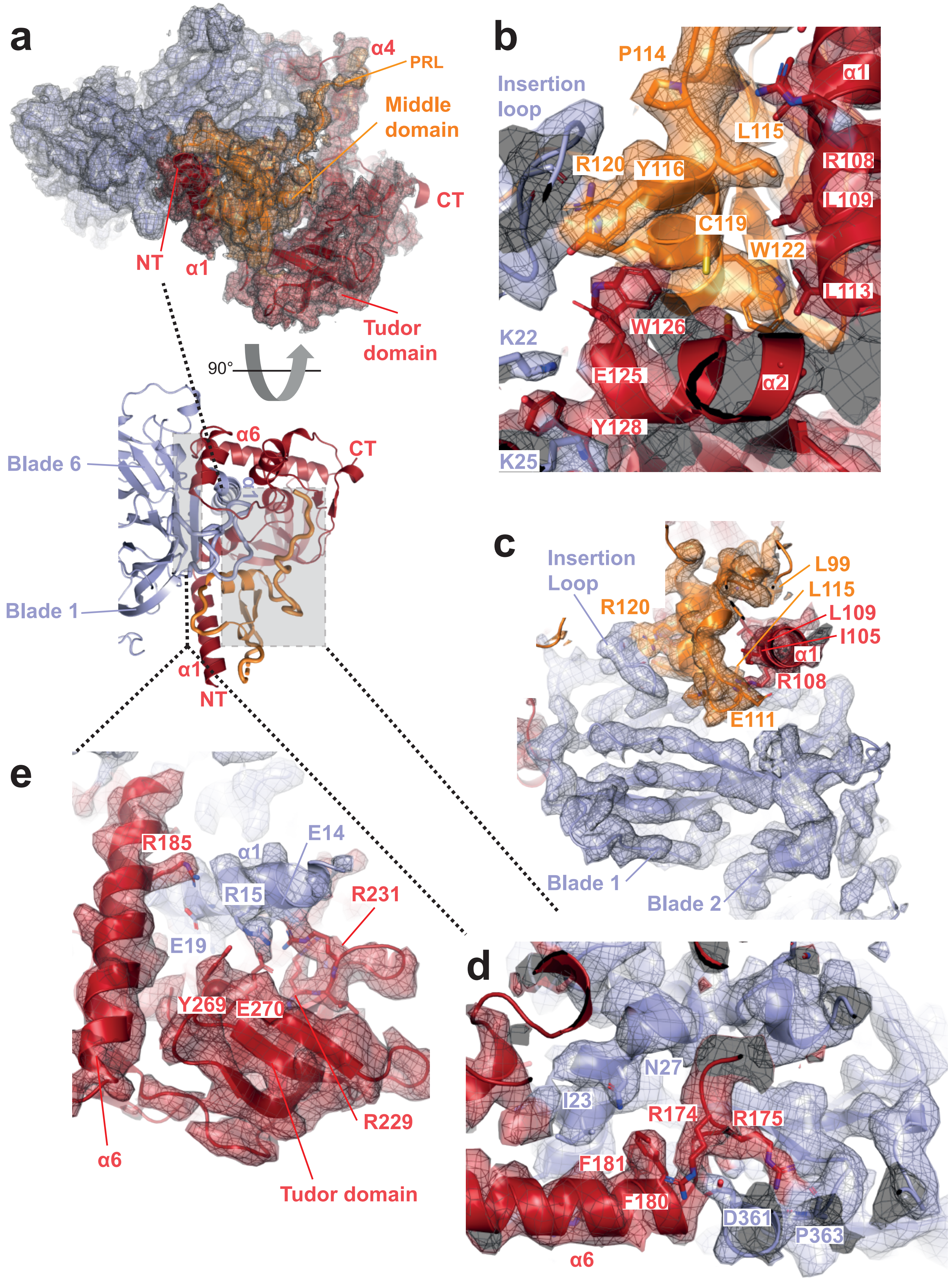

Supplementary Fig. 9. Cryo-EM structure and map of the MuvB apo complex. a-d Details of the cryo-EM map of the MuvB complex reconstruction are shown and are described in the main text.

Supplementary Figure 10

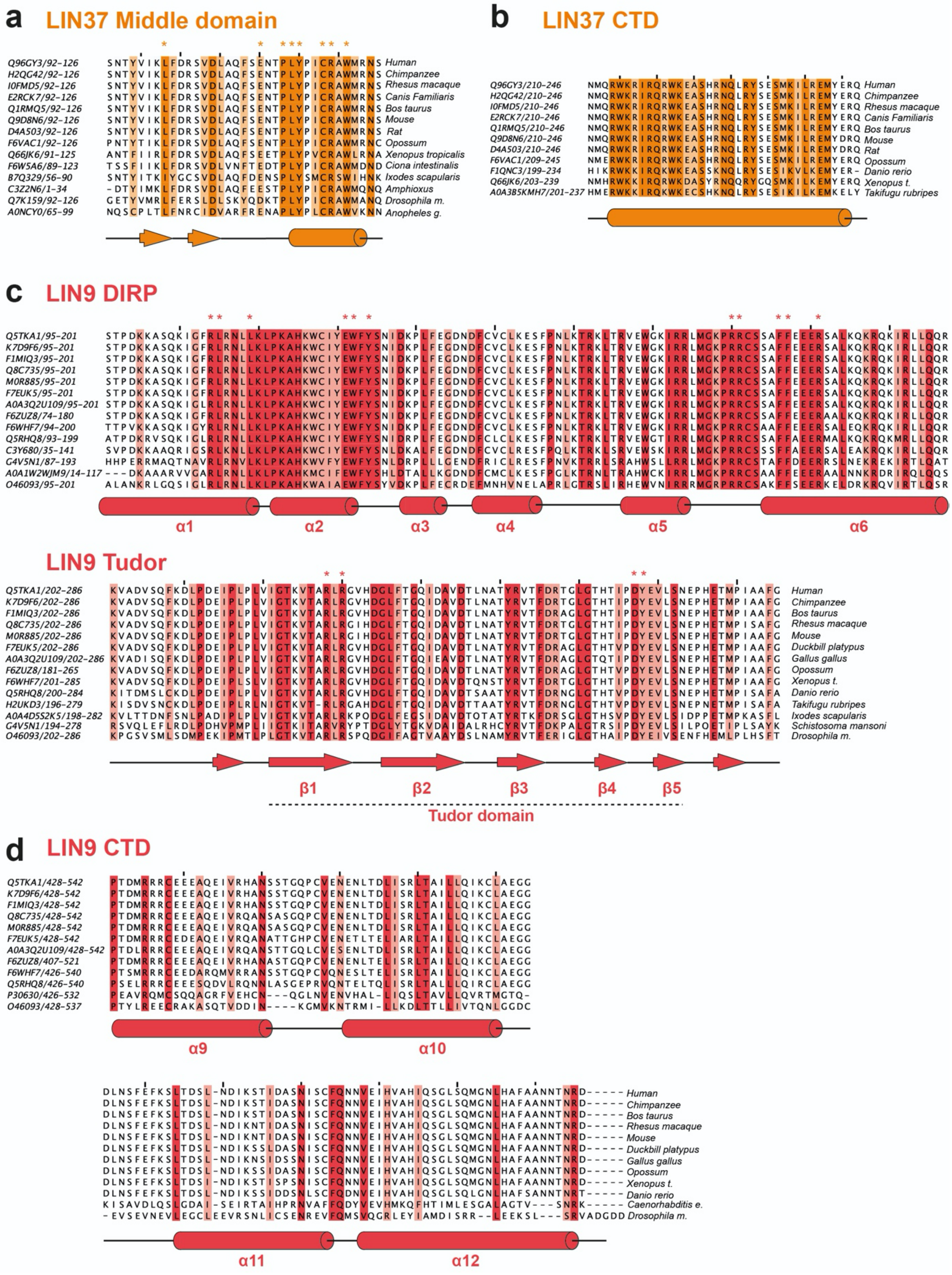

Supplementary Fig. 10. Conservation analysis on subunits of the MuvB complex. a-d Sequence alignments showing conservation of region of interest within this work.

Supplementary Table 1. Cryo-EM data collection, refinement and validation statistics

	MuvB-apo	MMB-HEAD	MMB-TAIL:NCP	LIN9CTD:NCP	MMBcore:DNA	NCP 127	NCP 147	MMBcore:NCP
**Data collection and processing**								
Magnification	240,000	165,000	165,000	165,000	165,000	165,000	165,000	165,000
Voltage (kV)	200	300	300	300	300	300	300	300
Electron exposure (e–/Å2)	60	60	60	60	60	60	60	60
Defocus range (μm)	-0.6 to -1.6	-0.6 to -1.6	-0.6 to -1.6	-0.6 to -1.6	-0.6 to -1.6	-0.6 to -1.6	-0.6 to -1.6	-0.6 to -1.6
Pixel size (Å)	0.567	0.513	0.513	0.513	0.513	0.513	0.513	0.513
Symmetry imposed	C1	C1	C1	C1	C1	C1	C1	C1
Initial particle images (no.)	597,933	648,272	648,272	648,272	648,272	648,272	648,272	648,272
Final particle images (no.)	26,641	23,399	23,399	237,061	51,225	20,934	11,330	23,399
Map resolution (Å)	3.5	7.5	8.7	7.1	7.5	3.3	6.9	10
FSC threshold	0.143	0.143	0.143	0.143	0.143	0.143	0.143	0.143
**Refinement**								
Initial model used (PDB code)	4pc0	-	-	-	-	-	-	-
Model resolution (Å)	3.5	-	-	-	-	-	-	-
Map sharpening B factor (Å2)	-66	-	-	-	-	-	-	-
Model composition								
Nonhydrogen atoms	5186	-	-	-	-	-	-	-
Protein residues	653	-	-	-	-	-	-	-
Ligands	-	-	-	-	-	-	-	-
B factors (Å2)								
Protein	94.27	-	-	-	-	-	-	-
R.m.s. deviations								
Bond lengths (Å)	0.005	-	-	-	-	-	-	-
Bond angles (°)	0.788	-	-	-	-	-	-	-
**Validation**								
MolProbity score	2.2	-	-	-	-	-	-	-
Clashscore	17.74	-	-	-	-	-	-	-
Poor rotamers (%)	0	-	-	-	-	-	-	-
Ramachandran plot								
Favored (%)	92.87	-	-	-	-	-	-	-
Allowed (%)	7.13	-	-	-	-	-	-	-
Disallowed (%)	0	-	-	-	-	-	-	-
