# Supplementary material for: Structure of a nucleosome-bound MuvB transcription factor complex reveals DNA remodelling
Source: Nat Commun. 2022 Aug 29;13:5075. doi: 10.1038/s41467-022-32798-9 (PMC9424243; doi:10.1038/s41467-022-32798-9)
Supplement: Supplementary file 3 — Description of additional Supplementary File [file 41467_2022_32798_MOESM3_ESM.pdf]

Descriptions of Additional Supplementary Data files

Supplementary Movie 1. MMB cryo-EM reconstructions and model fitting.

Supplementary Data 1. Crosslinking mass spectrometry data showing detected crosslinked peptides.
